# Gründe für allergologische Diagnostik und deren Ergebnisse im Kontext von COVID-19-Vakzinierungen: 2-jährige Erfahrung eines Allergiezentrums in Nordrhein-Westfalen

**DOI:** 10.1007/s00105-023-05152-3

**Published:** 2023-05-04

**Authors:** Eleni Leonidou Floruß, Anastasiia Demidova, Silke C. Hofmann, Galina Balakirski

**Affiliations:** Zentrum für Dermatologie, Allergologie und Dermatochirurgie, Helios Universitätsklinikum Wuppertal, Universität Witten/Herdecke, Heusnerstr. 40, 42283 Wuppertal, Deutschland

**Keywords:** COVID-19-Impfstoff, mRNA-Impfstoff, Impfreaktion, Allergie, Allergische Reaktion, Allergietestung, COVID-2019 vaccine, mRNA vaccine, Vaccination reaction, Allergy, Allergic reaction, Allergy testing

## Abstract

**Einleitung:**

Um der COVID-19-Pandemie entgegenzuwirken, wurden seit Dezember 2020 mehrere SARS-CoV-2-Impfstoffe zugelassen. Kurz nach Beginn der Impfkampagnen wurden einzelne allergische Reaktionen nach Vakzination beschrieben, was zu Unsicherheit vieler Patienten mit positiver allergologischer Anamnese geführt hat. Ziel dieser Arbeit war es zu erfassen, welche anamnestischen Ereignisse als Anlass für eine allergologische Abklärung vor COVID-19-Impfung dienten und welche Ergebnisse die entsprechende allergologische Diagnostik geliefert hat.

**Methoden:**

Es erfolgte eine retrospektive Datenanalyse aller Patienten, die sich während der Jahre 2021 und 2022 im Zentrum für Dermatologie, Allergologie und Dermatochirurgie des Helios Universitätsklinikums Wuppertal zur allergologischen Beratung oder Abklärung vor COVID-19-Vakzinierung vorstellten. Es wurden demografische Daten, allergologische Anamnese, Anlass der Konsultation in der Klinik und Ergebnisse allergologischer Diagnostik inklusive eventueller Reaktionen nach durchgeführter Impfung erfasst.

**Ergebnisse:**

Insgesamt stellten sich 93 Patienten zur allergologischen Abklärung in Bezug auf COVID-19-Vakzine vor. In etwa der Hälfte der Fälle erfolgte die Vorstellung in der Klinik aus Unsicherheit und Sorge vor allergischen Reaktionen und generellen Nebenwirkungen. Darunter waren 26,9 % (25/93) Patienten, die bisher noch keine COVID-19-Impfung erhalten hatten, und weitere 23,7 % (22/93), die eine nichtallergische Reaktion nach der bereits erfolgten COVID-19-Impfung (z. B. Kopfschmerzen, Schüttelfrost, Fieber, Unwohlsein etc.) erlitten haben; 46,2 % (43/93) der Patienten wurden aufgrund einer komplexen allergologischen Vorgeschichte in der Klinik geimpft. Dabei traten keine allergischen Reaktionen auf. Lediglich 1 Patientin mit bekannter chronisch-spontaner Urtikaria entwickelte mehrere Stunden nach Impfung ein Angioödem der Lippen, welches wir als nichtallergische Exazerbation der Grunderkrankung werteten. Den verbliebenen 53,8 % (50/93) der Patienten wurde eine ambulante Impfung in der Hausarztpraxis empfohlen.

**Diskussion:**

Allergische Reaktionen nach COVID-19-Impfstoffen sind selten, viele Patienten mit positiver allergologischer Anamnese haben jedoch Sorgen, nach der COVID-19-Vakzination allergisch zu reagieren. Öffentlichkeitsarbeit im Rahmen von Impfkampagnen durch allergologisch tätige Ärzte ist notwendig, um den Sorgen und Ängsten der Bevölkerung – und insbesondere Patienten mit Allergien in der Anamnese – gerecht zu werden.

## Abstract (en)

**Background:**

To counteract the coronavirus disease 2019 (COVID-19) pandemic, several severe acute respiratory syndrome coronavirus type 2 (SARS-CoV-2) vaccines have been licensed since December 2020. Shortly after the start of the vaccination campaigns, occasional allergic reactions related to vaccines were described, thus, leading to concerns in many patients with a history of allergies. The aim of this work was to evaluate which anamnestic events represented a reason for an allergology work-up before COVID-19 vaccination. Furthermore, the results of the allergology diagnostics are described.

**Methods:**

We performed a retrospective data analysis of all patients who presented at the Center for Dermatology, Allergology and Dermatosurgery of the Helios University Hospital Wuppertal during the years 2021 and 2022 for allergology work-up prior to COVID-19 vaccination. Demographic data, allergological history, reason for the consultation in the clinic and results of allergology diagnostic tests including reactions after vaccination were included.

**Results:**

A total of 93 patients presented for allergology work-up with COVID-19 vaccines. In about half of the cases, the reasons for the presentation to the clinic were doubts and concerns about allergic reactions and side effects. In all, 26.9% (25/93) of the presented patients had not previously received a COVID-19 vaccine yet and 23.7% (22/93) of patients developed non-allergic reactions after prior COVID-19 vaccine (e.g., headache, chills, fever, malaise). Of the patients, 46.2% (43/93) were successfully vaccinated in the clinic due to a complex allergological history, while the remaining 53.8% (50/93) were subjected to outpatient vaccination in the vaccination practice. Only one patient with known chronic spontaneous urticaria developed a mild angioedema of the lips a few hours after vaccination; however, we do not consider this episode an allergic reaction to the vaccine due to the time delay.

**Conclusion:**

Allergic reactions after COVID-19 vaccines are rare, but many patients with a positive medical history of allergies are concerned about allergic reactions after COVID-19 vaccination. Thus, public work by practicing allergologists is important during vaccination campaigns to meet the concerns and fears of the population, especially of patients with allergies in their medical history.

Mit dem ersten bestätigten Fall am 27.01.2020 brach eine SARS-CoV-2-Epidemie auch in Deutschland aus, und bereits am 11.03.2020 erklärte die Weltgesundheitsorganisation (WHO) den COVID-19-Ausbruch weltweit zu einer Pandemie [1]. Um der COVID-19-Pandemie entgegenzuwirken, wurden seit Dezember 2020 mehrere SARS-CoV-2-Impfstoffe zugelassen [2]. Kurz nach Beginn der Impfkampagnen wurden einzelne allergische Reaktionen nach Vakzination beschrieben, was zu Unsicherheit vieler Patienten mit positiver allergologischer Anamnese führte [3, 4]. So veröffentlichten die deutschen allergologischen Gesellschaften AeDA (Ärzteverband Deutscher Allergologen), DGAKI (Deutsche Gesellschaft für Allergologie und klinische Immunologie) und GPA (Gesellschaft für Pädiatrische Allergologie und Umweltmedizin) eine Stellungnahme bezüglich schwerer allergischer Reaktionen nach COVID-19-Impfung, die in den USA und Großbritannien beobachtet wurden [5]. Später wurde auch eine Patienteninformation diesbezüglich erstellt [6]. Nach dem aktuellen Wissensstand kommen die beschriebenen allergischen Reaktionen auf COVID-19-Vakzinen insgesamt selten vor. Die Patienten mit früherer schwerer allergischer Reaktion auf Inhaltsstoffe des Impfstoffes (z. B. Macrogol) oder auf die erste COVID-19-Impfung sollten ähnlich wie die Patienten mit früherer schwerer allergischer Reaktion nach Arzneimitteln bei bekannter Mastozytose oder schwerer allergischer Reaktion unbekannter Ursache eine allergologische Abklärung vor der geplanten SARS-CoV-2-Impfung erhalten. Das Gleiche wurde empfohlen für Patienten nach schwerer allergischer Reaktion bei einer früheren Nicht-COVID-19-Impfung [7]. Für Patienten mit bekannten Arzneimittelallergien, Insektengiftallergien, Nahrungsmittelallergien oder Erkrankungen aus dem atopischen Formenkreis wie atopisches Ekzem, allergische Rhinitis und allergisches Asthma wurden ebenso wie für Patienten ohne allergologische Vorgeschichte keine Hinweise für ein erhöhtes Risiko im Zusammenhang mit einer COVID-19-Impfung gesehen [6, 7].

Obwohl die Impfbereitschaft gegen COVID-19 in Deutschland durch das Robert-Koch-Institut als gut bewertet wurde, stellte die COVIMO-Befragung (COVID-19 Impfquoten-Monitoring in Deutschland) fest, dass auch noch 1 Jahr nach Beginn der Impfaktivitäten viel Unsicherheit um die COVID-19-Impfungen bestand. So konnte nur ein Drittel der Umfrageteilnehmer (33,7 %) auf die Fragen, ob COVID-19-Impfung Allergien verursacht, richtig antworten [8]. Es bleibt daher unklar, ob trotz der oben genannten Empfehlungen der allergologischen Fachgesellschaften Patienten mit allergologischer Vorgeschichte oder Atopieanamnese die COVID-19-Impfungen aus Angst und Sorge vor möglichen allergischen Reaktionen ablehnten. Ziel dieser Arbeit war zu erfassen, welche anamnestischen Ereignisse als Anlass für eine allergologische Vorstellung vor COVID-19-Impfung dienten und welche Ergebnisse die entsprechende allergologische Diagnostik geliefert hat.

## Methoden

### Datenerhebung

Es erfolgte eine retrospektive Analyse der Patientenakten aller Patienten, die sich im Zeitraum vom 01.01.2021 bis zum 31.12.2022 (24 Monate) im Zentrum für Dermatologie, Allergologie und Dermatochirurgie des Helios Universitätsklinikums Wuppertal zwecks allergologischer Diagnostik auf COVID-19-Impfstoffe vorstellten. Die Klinik verfügt über eine fachallergologische Sprechstunde und ein multidisziplinäres Team (Bergisches Allergiezentrum). Nur die Patienten, für die vollständige klinische Dokumentation und Aufzeichnung der durchgeführten allergologischen Testungen auf Bestandteile der SARS-CoV-2-Impfstoffe oder Impfstoffe selbst vorlagen, wurden in die Auswertung eingeschlossen.

Die aus den Patientenakten extrahierte Information umfasste Alter und Geschlecht der Patienten, die ausführliche allergologische Anamnese, Zeitpunkt (Monat und Jahr) der Vorstellung in der Klinik, Gründe für die angestrebte allergologische Testung auf COVID-19-Impfstoffe und anamnestische Reaktionen auf COVID-19-Impfstoffe, falls eine entsprechende Impfung vorausgegangen war. Im Falle einer vorausgegangenen Reaktion auf einen COVID-19-Impfstoff wurde die Symptomatik bzw. Art der Reaktion miterfasst.

Die allergologische Anamnese beinhaltete vorbekannte Arzneimittelallergien vom Sofort- oder Spättyp, Nahrungsmittelallergien, Insektengiftallergien, Erkrankungen des atopischen Formenkreises (atopisches Ekzem, allergisches Asthma, [Rhino-]Conjunctivitis allergica), chronisch-spontane oder induzierbare Urtikaria, allergische Kontaktsensibilisierungen, kutane oder systemische Mastozytose, hereditäres Angioödem, Anaphylaxien unklarer Genese und persistierende Tryptaseerhöhung im Serum ohne nachweisbare Mastozytose.

Für das Forschungsvorhaben lag ein positives Ethikvotum der lokalen Ethikkommission vor (S-200/2021, Ethik-Kommission der Universität Witten/Herdecke).

### Allergologische Diagnostik

Neben der Erfassung demografischer Daten und der allergologischen Anamnese erfolgte die Analyse der durchgeführten allergologischen Diagnostik.

Es erfolgte eine In-vivo-Diagnostik mittels einer Hautpricktestung. Folgende Testsubstanzen wurden angewendet: Polysorbat 80 1 % in wässriger Lösung (pur), Polysorbat 80 10 % in wässriger Lösung (pur), Macrogol (Polyethylenglycol) 1500 (pur), Macrogol (Polyethylenglycol) 4000, Trometamol (pur), Comirnaty®-Impfstoff (BioNTech/Pfizer) (pur) und Spikevax®-Impfstoff (Moderna) (pur). Die Testung für Macrogol (Polyethylenglycol) 2000 als Reinsubstanz wurde durch die Testung mit den Impfstoffen ersetzt. Der Umfang der durchgeführten Hautpricktestung variierte in Abhängigkeit von den zum Zeitpunkt der Testung verfügbaren Testsubstanzen.

Die Hautpricktestungen erfolgten an den Unterarminnenseiten und wurden gemäß den nationalen und internationalen Empfehlungen durchgeführt [9, 10]. Histamindihydrochlorid (10 mg/ml) wurde als Positivkontrolle und isotone Kochsalzlösung als Negativkontrolle angewendet. Die Ablesung erfolgte nach 20 Minuten. Eine Testquaddel mit einem Durchmesser von mindestens 3 mm wurde als positiv bewertet.

Im Falle einer Provokationstestung wurde die gesamte Impfdosis ohne Fraktionierung intramuskulär in den Oberarm verabreicht. Die anschließende Überwachung der geimpften Patienten betrug mindestens 2 Stunden.

### Statistische Analyse

Die statistische Analyse und die grafische Darstellung der Daten erfolgte mittels Microsoft Excel 2016 für Windows.

Die Verteilung der kategorischen (nominalen) Daten wurde durch absolute und relative Häufigkeiten dargestellt und mittels Chi-Quadrat-Tests (χ^2^-Test) zwischen den Gruppen verglichen. Die statistische Signifikanz wurde mit einem zweiseitigen Hypothesentest auf einem Signifikanzniveau von 5 % berechnet. Deskriptive Statistik von metrisch skalierten Variablen wurde mittels Mittelwert und Standardabweichung (±SD) dargestellt. Für den Vergleich der Mittelwerte zwischen den Gruppen wurde der t‑Test verwendet.

## Ergebnisse

### Demografische und klinische Patientencharakteristiken

Im Zeitraum zwischen 01.01.2021 und 31.12.2022 stellten sich 93 Patienten im Bergischen Allergiezentrum mit der Frage nach einer allergologischen Abklärung in Bezug auf die COVID-19-Impfung vor. Davon waren 77,4 % (72/93) weiblich. Das mittlere Alter aller Patienten betrug 49,7 ± 16,4 Jahre. Das mittlere Alter weiblicher Patientinnen (49,9 ± 16,6) unterschied sich nicht vom mittleren Alter männlicher Patienten (48,9 ± 16,1; *p* = 0,81).

Es erfolgten 81,7 % (76/93) aller allergologischen Konsultationen im Jahr 2021, davon knapp die Hälfte (48,7 %; 37/76) im 3. Quartal des Jahres (Tab. 1).

**Table 1:** 

Monat	Jahr	
	2021	2022
Januar	0	4
Februar	0	4
März	1	2
April	7	3
Mai	8	1
Juni	7	0
Juli	13	2
August	17	0
September	7	0
Oktober	7	1
November	5	0
Dezember	7	0
Gesamt	76	17

### Allergologische Vorgeschichte

Es berichteten 55,9 % (52/93) der Patienten (davon 41 Frauen und 11 Männer) über eine bekannte allergologische Vorgeschichte ohne einen Zusammenhang mit Bestandteilen der COVID-19-Impfstoffe oder Reaktionen auf die COVID-19-Impfung (Tab. 2). Davon gaben 65,4 % (34/52) mehr als eine allergologische Diagnose an (2 oder mehr unterschiedliche Allergiearten, beispielsweise Nahrungsmittelallergie und Arzneimittelallergie, o. Ä.).

**Table 2:** 

Allergologische Anamnese	Geschlecht		
	Weiblich (*n*/%)	Männlich (*n*/%)	Gesamt (*n*/%)
Arzneimittelallergie vom Soforttyp	37/51,3	8/38,1	45/48,3
Arzneimittelallergie vom Spättyp	4/5,6	0	4/4,3
Nahrungsmittelallergie	9/12,5	1/4,8	10/10,8
Allergische Rhinitis	28/38,9	10/47,6	38/40,9
Allergisches Asthma	9/12,5	3/14,3	12/12,9
Atopisches Ekzem	4/5,6	3/14,3	7/7,5
Insektengiftallergie	6/8,3	1/4,8	7/7,5
Histaminintoleranz	3/4,2	1/4,8	4/4,3
Anaphylaxie unklarer Genese	2/2,8	1/4,8	3/3,2
Chronisch-induzierbare Urtikaria	6/8,3	1/4,8	7/7,5
Chronisch-spontane Urtikaria	3/4,2	0	3/3,2
Hereditäres Angioödem	1/1,4	0	1/1,1
Persistierende Tryptaseerhöhung ohne nachweisbare Mastozytose	1/1,4	0	1/1,1
Kutane oder systemische Mastozytose	9/12,5	0	9/9,7
Allergische Kontaktallergie	18/25	4/19,0	21/22,6
Allergie gegenüber anderen Impfstoffen	3/4,2	2/9,5	5/5,4

### Impfstatus der Patienten und Reaktion auf vorherige COVID-19-Impfungen

Vor der Vorstellung im Bergischen Allergiezentrum wurden 37,6 % (35/93) der Patienten bereits mindestens einmalig gegen COVID-19 auswärts geimpft. Es handelte sich dabei um 36,1 % (26/72) Frauen und 42,8 % (9/21) Männer. Die Impfrate männlicher Patienten zeigte keinen statistisch signifikanten Unterschied im Vergleich zur Impfrate weiblicher Patientinnen (*p* = 0,57).

Es hatten 71,4 % (25/35) der bereits zum Zeitpunkt der Vorstellung gegen COVID-19 vakzinierten Patienten eine einzelne Impfdosis, 20,0 % (7/35) 2 Impfdosen und die restlichen 8,6 % (3/35) 3 Impfdosen bereits erhalten; 85,7 % (30/35) wurden dabei mit dem Comirnaty®-Impfstoff (BioNTech/Pfizer), 11,4 % (4/35) mit dem Vaxzevria®-Impfstoff (AstraZeneca) und 2,8 % (1/35) mit dem Spikevax®-Impfstoff (Moderna) geimpft.

Es hatten 94,3 % (33/35) der bereits gegen COVID-19 geimpften Patienten, die sich zur allergologischen Testung vorstellten, anamnestisch nach der vorangegangenen Impfung Reaktionen entwickelt, die nun abgeklärt werden sollten. Die restlichen 5,7 % (2/35) der Patienten hatten zwar die erfolgten Vakzinierungen mit einem Vektorimpfstoff problemlos vertragen, sollten allerdings als Booster-Impfung einen mRNA-Impfstoff erhalten und wegen möglicher Sensibilisierung gegen Macrogol in der Vorgeschichte vor der geplanten Impfung abgeklärt werden.

**Figure 1:**
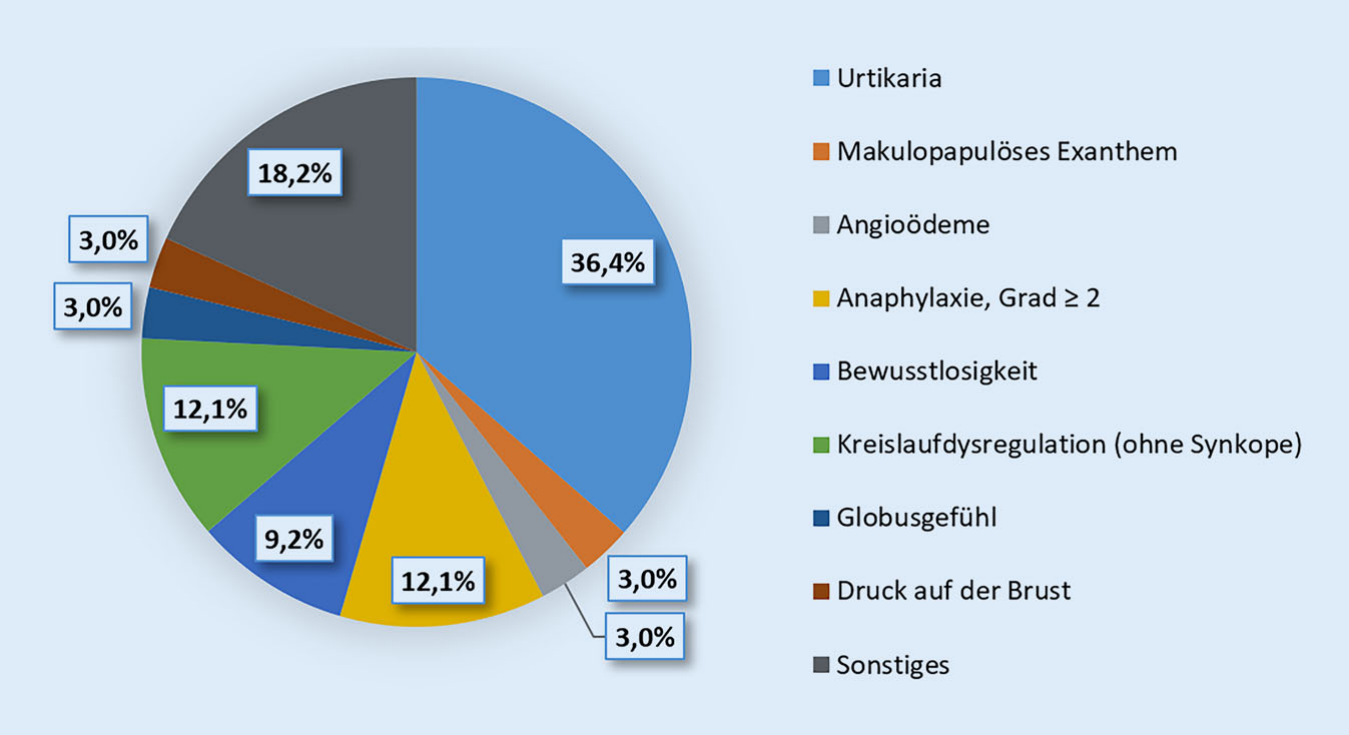

Die häufigsten nach einer COVID-19-Impfung berichteten Reaktionen stellten mit jeweils 36,4 % (12/33) Urtikaria, 21,3 % (7/33) Kreislaufdysregulation oder Synkope und 12,1 % (4/33) Anaphylaxie (Grad ≥ 2) dar. Zudem sind in 18,2 % (6/33) der Fälle sonstige Symptomkomplexe (Reizhusten, Diarrhö, Übelkeit, Schweißausbrüche, Kopfschmerzen, Lähmungsgefühl, Flush-Symptomatik, allgemeines Unwohlsein, o. Ä.) aufgetreten (Abb. 1).

### Gründe für die Vorstellung zur allergologischen Abklärung

Es haben sich 11,8 % (11/93) der Patienten aufgrund allergologischer Symptomatik (wie Urtikaria, Angioödeme, Dyspnoe oder Kreislaufdysregulation) innerhalb 60 Minuten nach vorangegangener COVID-19-Impfung zur Abklärung vorgestellt; 23,7 % (22/93) der Patienten strebten eine allergologische Abklärung aufgrund fraglich allergologischer (z. B. Urtikaria nach > 24 Stunden) oder nichtallergischer Symptomatik (wie Unwohlsein, Schwindel, Kopfschmerzen, Diarrhöen, Übelkeit o. Ä.) nach einer COVID-19-Vakzination an.

Es berichteten 8,6 % (8/93) der Patienten über eine allergische Typ-I- oder Typ-IV-Sensibilisierung gegenüber Macrogol als Grund für die allergologische Vorstellung. In 5,4 % (5/93) der Fälle wurde über eine allergische Reaktion nach einer anderen Impfung berichtet. Bei 26,8 % (25/93) der Patienten waren Arzneimittelallergien oder andere Allergien bekannt, sodass lediglich Unsicherheit bezüglich des COVID-19-Impfstoffs seitens der Patienten bestand. Es ergab sich kein statistisch signifikanter Unterschied zwischen Frauen und Männern in Bezug auf den Anlass zur allergologischen Vorstellung (Tab. 3).

**Table 3:** 

Grund für die Vorstellung	Geschlecht		
	Weiblich (*n*/%)	Männlich (*n*/%)	p‑Wert
Allergische Reaktion nach vorangegangener COVID-19-Impfung	9/12,5	2/9,5	0,85
Nichtallergische Reaktion nach vorangegangener COVID-19-Impfung	17/23,6	5/23,8	0,99
Allergische Reaktion nach anderer Impfung	2/2,8	3/14,3	0,052
Nichtallergische Reaktion nach anderer Impfung	3/4,2	0	0,35
Soforttypallergie gegenüber Macrogol	2/2,8	0	0,45
Spättypallergie gegenüber Macrogol	4/5,5	2/9,5	0,53
Kutane oder systemische Mastozytose	9/12,5	0	0,09
Allergische Reaktionen unklarer Genese	0	1/4,76	0,06
Schwere allergische Reaktion nach Medikamenteneinnahme in Vergangenheit	6/8,3	3/14,3	0,44
Angst und Unsicherheit	20/27,7	5/23,8	0,76

### Ergebnisse allergologischer Abklärung

Bei 92 (98,9 %) Patienten wurde eine Hautpricktestung zwecks allergologischer Abklärung durchgeführt; 90,2 % (83/92) der Patienten wurden gegenüber Macrogol 1500 und/oder 4000 getestet, 13,0 % (12/92) der Patienten wurden gegenüber Polysorbat 80 getestet. Des Weiteren wurde in 15,2 % (15/92) der Fälle eine Hautpricktestung mit Trometamol durchgeführt. Zudem erfolgten bei 78,3 % (72/92) und 9,8 % (9/92) der Patienten Pricktests mit jeweils Comirnaty®-Impfstoff (BioNTech/Pfizer) und Spikevax®-Impfstoff (Moderna). In 93,6 % (87/93) der Fälle ergab sich im Rahmen der allergologischen Abklärung kein Hinweis auf das Vorliegen einer allergischen Typ-I-Sensibilisierung gegenüber den getesteten COVID-19-Impfstoffen oder ihren Inhaltsstoffen. Bei 3,3 % (3/92) der Patienten war die Hautpricktestung aufgrund einer Urticaria factitia nicht beurteilbar. Zwei der letzteren Patienten wurden in der Klinik erfolgreich geimpft. Bei dem weiteren Patienten wurde aufgrund einer unauffälligen allergologischen Vorgeschichte in Bezug auf COVID-19-Impfstoffe eine ambulante Impfung in der Hausarztpraxis empfohlen. Bei den restlichen 3,3 % (3/92) ergab sich im Hautpricktest eine positive Reaktion. Davon zeigte 1 Patient eine schwach positive Testreaktion auf einen mRNA-Impfstoff, konnte aber dennoch anschließend erfolgreich in der Klinik geimpft werden. Zwei weitere Patienten zeigten eine positive Reaktion auf Macrogol und den COVID-19-Impfstoff im Pricktest. Da einer dieser Patienten bisher keine COVID-19-Impfung erhalten hatte, wurde er in Notfallbereitschaft mit einem mRNA-Impfstoff geimpft und 24 Stunden stationär nachbeobachtet. Die Impfung wurde problemlos vertragen. Der andere Patient berichtete über eine allergische Reaktion (Anaphylaxie Grad 2 mit Ganzkörpererythem, Dyspnoe, Übelkeit und Kreislaufdysregulation) wenige Minuten nach Verabreichung der COVID-19-mRNA-Vakzine, sodass die weitere geplante Impfung mit einem Vektorimpfstoff in Notfallbereitschaft empfohlen wurde. Bei einem Patienten wurde keine Hautpricktestung durchgeführt. Hierbei handelte es sich um einen Patienten mit chronisch-spontaner Urtikaria, der in der Klinik direkt erfolgreich geimpft werden konnte.

Insgesamt wurden 46,2 % (43/93) der Patienten aufgrund einer komplexen allergologischen Vorgeschichte in der Klinik geimpft, den restlichen 53,8 % (50/93) der Patienten wurde eine ambulante Impfung in der Hausarztpraxis empfohlen; 51,2 % (22/43) der im Bergischen Allergiezentrum geimpften Personen erhielten in der Klinik die Erstimpfung mit COVID-19-Impfstoff, wobei 18,2 % (4/22) der Personen darüber hinausgehend auch für die 2. und/oder 3. Impfung erneut in die Klinik eingewiesen wurden bei komplexer allergologischer Vorgeschichte.

**Figure 2:**
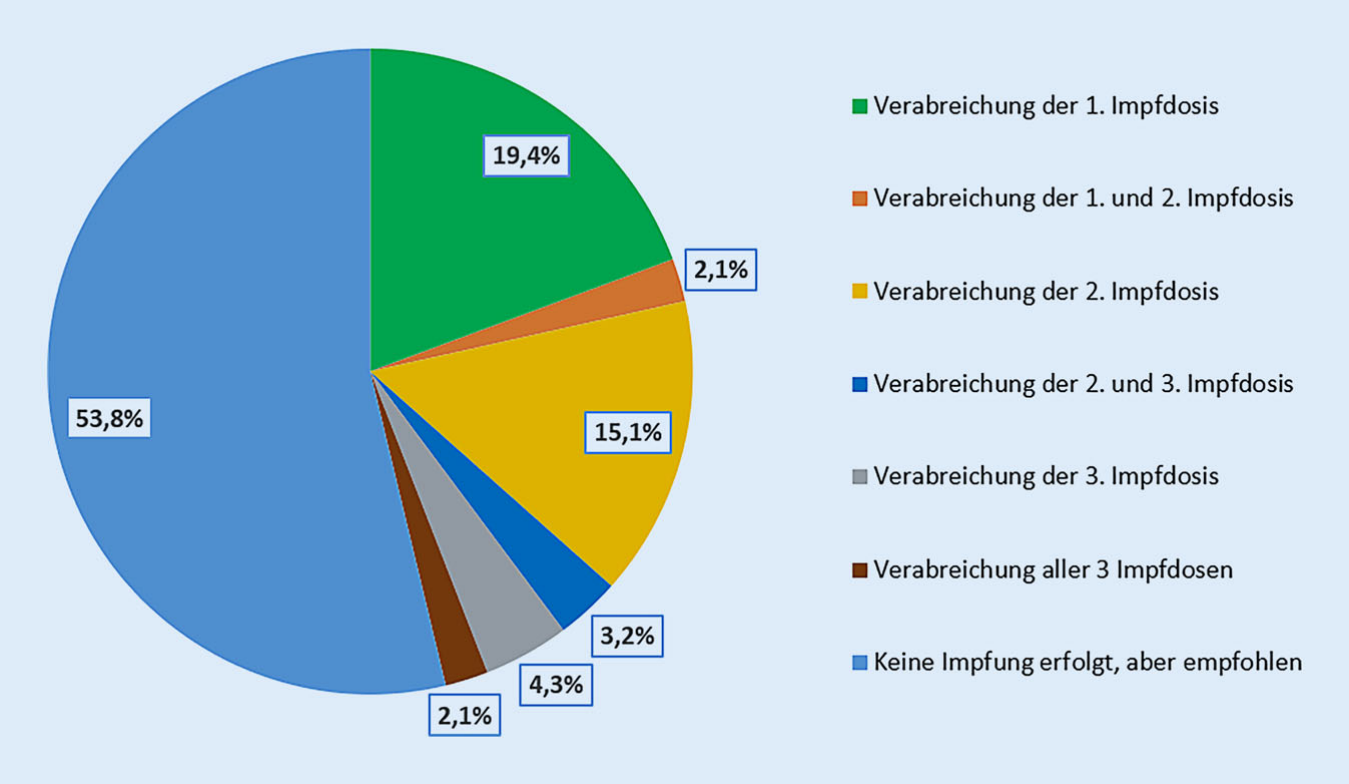

Die restlichen 48,8 % (21/43) der in der Klinik geimpften Personen erhielten vor der Vorstellung in der Klinik mindestens 1‑malig eine COVID-19-Impfung; 66,7 % (14/21) dieser Patienten wurden im Bergischen Allergiezentrum mit der 2. Impfdosis geimpft, 14,3 % (3/21) und 19,0 % (4/21) der Patienten erhielten jeweils die 2. und 3. bzw. nur die 3. Impfdosis (Abb. 2).

Insgesamt wurden somit 52 Impfdosen verabreicht, davon 55,8 % (29/52) ambulant und 44,2 % (23/52) unter stationären Bedingungen. In 94,2 % (49/52) der Fälle handelte es sich um Comirnaty®-Impfstoff (BioNTech/Pfizer), die restlichen 5,8 % (3/52) Vakzinationen erfolgten mit Spikevax®-Impfstoff (Moderna). In 90,6 % (39/43) der Fälle wurde die Impfung problemlos vertragen; 2,3 % (1/43) der geimpften Patienten reagierten mit jeweils Blutdruckanstieg, vorübergehendem Globusgefühl und Exazerbation eines bekannten Asthma bronchiale. In 1 Fall (2,3 %; 1/43) kam es bei einer Patientin mit bekannter chronisch-spontaner Urtikaria zu einem milden Angioödem der Lippen am Tag nach der Impfung, welches nach oraler Gabe eines Antihistaminikums rückläufig war. Schwere allergische Reaktionen sind nicht aufgetreten.

## Diskussion

Allergische Reaktionen nach Verabreichung der COVID-19-Impfstoffe sind selten. Die Prävalenz liegt bei 7,91 Fällen auf 1 Mio. Impfdosen in der Allgemeinbevölkerung [11]. Auch in den Zulassungsstudien wurden allergische Reaktionen, Urtikaria oder Angioödeme mit ähnlicher Häufigkeit nach der Vakzinierung und im Placeboarm beobachtet [12, 13]. Des Weiteren konnte gezeigt werden, dass Patienten mit hereditärem Angioödem ohne schwerwiegende Ereignisse mit COVID-19-Impfstoffen geimpft werden können [14].

Die aktuellen Empfehlungen der allergologischen Fachgesellschaften beinhalten eine Einschätzung bezüglich des Risikos für eine allergische Reaktion nach der COVID-19-Impfstoff-Gabe. Dabei haben Patienten mit atopischem Ekzem und Erkrankungen des atopischen Formenkreises, chronisch-spontaner Urtikaria, Insektengiftallergie, Nahrungsmittelallergien, Antibiotikaallergien oder Analgetikaintoleranz im Vergleich zur Allgemeinbevölkerung kein erhöhtes Risiko, nach der Verabreichung eines COVID-19-mRNA-Impfstoffs eine allergische Reaktion zu erleiden und können deswegen ohne weitere allergologische Abklärung geimpft werden [5–7]. Eine allergologische Abklärung wird nur für besondere Patientengruppen empfohlen. So wird es gemäß den oben genannten Empfehlungen angeraten, folgenden Patienten eine allergologische Abklärung anzubieten: bei schweren allergischen Reaktionen nach früherer Nicht-COVID-19-Impfung, gegenüber Medikamenten oder bei unklarer Genese sowie bei schweren allergischen Reaktionen nach Medikamentenverabreichung bei bekannter Mastozytose [5–7]. Auch bei einer Macrogol-Allergie in der Anamnese oder allergischen Symptomen nach erfolgter COVID-19-Impfung sollte vor der nächsten Impfdosis eine allergologische Diagnostik erfolgen [15]. Da die beiden in Deutschland zugelassenen mRNA-COVID-19-Impfstoffe Macrogol (Polyethylenglycol [PEG] 2000) enthalten, können Patienten mit früheren allergischen Reaktionen auf Macrogol-haltige Abführmittel auf die Gabe dieser Impfstoffe allergisch reagieren, was vorherige allergologische Diagnostik notwendig macht.

In der aktuellen wissenschaftlichen Literatur sind Hautpricktestungen mit PEG, Polysorbat 80 (welches in einigen Vektorimpfstoffen enthalten ist und gelegentlich eine Kreuzreaktivität mit PEG zeigt), Trometamol (welches im Spikevax®-Impfstoff von Moderna enthalten ist) und eigentlichen Impfstoffen beschrieben und empfohlen [16–19]. Es wurde zudem berichtet, dass auch Patienten, die allergische Symptome nach einer COVID-19-Impfung entwickelt und eine positive Hautreaktion im Rahmen der allergologischen Abklärung gezeigt haben, die 2. Gabe des gleichen Impfstoffs problemlos vertragen können [18, 20–23]. Als Grund für die positive Hautpricktestung wird eine unspezifische oder irritative Hautreaktion auf die getesteten Substanzen vermutet [18]. Insgesamt konnte bestätigt werden, dass COVID-19-Impfstoffe aus allergologischer Sicht sicher sind und die nachgewiesenen allergischen Typ-I-Reaktionen nach diesen Vakzinen nicht häufiger auftreten als nach anderen klassischen Impfstoffen [22, 23].

Die 2‑jährige Erfahrung aus dem Bergischen Allergiezentrum in Wuppertal lieferte ähnliche Ergebnisse. Von den 43 in der Klinik geimpften Patienten entwickelte eine Patientin mit chronisch-spontaner Urtikaria ein mildes Angioödem der Lippen einige Stunden nach der Vakzination. Dieses Ereignis interpretieren wir jedoch aufgrund der zeitlichen Verzögerung nicht als allergische Reaktion auf den Impfstoff, sondern gehen von einem Angioödem im Rahmen der bekannten Grunderkrankung aus. Vereinzelt traten subjektive Symptome (wie Kopfschmerzen, vorübergehend subjektiv erschwerte Atmung, etc.) nach der Impfung auf. Da eine fraktionierte Gabe des Impfstoffes und Verabreichung der gesamten Dosis bei der allergologischen Provokationstestung im Kontext der COVID-19-Impfung in der wissenschaftlichen Literatur als gleichwertig bewertet wurde [24], wurde in unserer Klinik eine direkte intramuskuläre Gabe der gesamten Dosis gewählt, um die meist ohnehin ängstlichen Patienten nicht mit mehreren Injektionen zu belasten. Dieses Vorgehen stellte sich im Laufe der Impftätigkeit in unserem Allergiezentrum als sicher und gut verträglich heraus.

Unsere Erfahrungen zeigen jedoch, dass etwa die Hälfte der Patienten, die eine allergologische Abklärung anstrebten, sich in der Klinik aus Unsicherheit und Sorge vor allergischen Reaktionen und generellen Nebenwirkungen vorgestellt hat. Dabei handelte es sich um 26,9 % (25/93) der Patienten, die bisher noch keine COVID-19-Impfung erhalten haben, und in 23,7 % (22/93) der Fälle um Patienten, die eine nichtallergische Reaktion nach der bereits erfolgten COVID-19-Impfung (z. B. Kopfschmerzen, Schüttelfrost, Fieber, Unwohlsein, etc.) erlitten haben. Ein Geschlechterunterschied wurde dabei nicht beobachtet. Da solche Unsicherheit der Patienten in vielen Fällen aus nicht ausreichender Information, Beratung und Aufklärung resultiert [25], ist es wichtig, aus der Erfahrung der COVID-19-Impfkampagne für zukünftige Impfkampagnen Konsequenzen zu ziehen. Auch die bereits erwähnte COVIMO-Umfrage des Robert Koch-Instituts zeigt, dass die deutsche Bevölkerung über die Sicherheit der COVID-19-Impfstoffe schlecht informiert ist [8]. Eine ähnliche Lage fand sich auch in den USA: Nach Zulassung der COVID-19-Impfstoffe für Jugendliche berichteten Eltern, die mit der Vakzinierung ihrer Kinder zögerten, dass mehr Information über die Sicherheit der Vakzine die Akzeptanz des COVID-19-Impfstoffs erhöhen würde [26, 27]. Für eine erfolgreiche Impfkampagne ist somit ein hohes Maß an Öffentlichkeitsarbeit und Aufklärung wichtig.

Da 81,7 % (76/93) der allergologischen Vorstellungen in unserer Klinik im Jahr 2021 erfolgten, ist es naheliegend, dass das Thema in den Augen der Patienten im Jahr 2022 an Aktualität verloren hat. Die Gründe dafür sind möglicherweise mildere COVID-19-Verläufe und geringere Mortalität an COVID-19, sodass eine COVID-19-Impfung vielen Patienten nicht mehr wichtig erschien, oder die Lockerung der Pandemie-bedingten Einschränkungen, sodass Reisen und Teilnahme an öffentlichen Veranstaltungen auch für ungeimpfte Personen möglich geworden ist. Zudem ist es möglich, dass durch das breite Impfangebot ab Mitte 2021 die meisten impfbereiten Personen bereits geimpft worden sind. Ein weiterer möglicher Grund für die Abnahme allergologischer Vorstellungen zur Testung mit COVID-19-Vakzinen ist ein verbesserter Informationsfluss über die hohe Sicherheit der Impfstoffe durch eine über 1 Jahr dauernde Impfkampagne. Wahrscheinlich haben alle hier dargestellten Faktoren dazu beigetragen, dass im 2. Halbjahr 2022 im Bergischen Allergiezentrum nur 3 Personen sich mit der Frage nach allergologischer Abklärung einer möglichen Unverträglichkeit der COVID-19-Impfstoffe vorgestellt haben.

Die Limitation unserer Daten liegt daran, dass das Bergische Allergiezentrum eine Ermächtigungsambulanz darstellt und nur Patienten, die von Fachärzten für Allergologie überwiesen werden, beraten und behandelt werden können. Zudem liegt keine Information über die Impfstofftoleranz der 53,8 % (50/93) Personen vor, bei denen die allergologische Abklärung unauffällig ausfiel und eine Vakzination ambulant (z. B. beim Hausarzt oder in einem Impfzentrum) empfohlen wurde. Generell liegen keine Daten dazu vor, wie viele Personen in Deutschland aufgrund von Sorgen vor allergischen Reaktionen nach COVID-19-Impfung die Vakzinierung abgelehnt haben. Am Beispiel der COVID-19-Vakzinierung konnte nun gezeigt werden, dass Bedarf für Öffentlichkeitsarbeit im Rahmen von Impfkampagnen durch allergologisch tätige Ärzte besteht, um den Sorgen und Ängsten der Bevölkerung – und insbesondere der Patienten mit Allergien in der Anamnese – gerecht zu werden. Bei begründeten Verdachtsfällen kann allerdings erst die allergologische Diagnostik Unsicherheiten ausräumen, sodass allergologisch tätige Praxen und Kliniken eine wichtige Säule der Patientenversorgung im Rahmen der Impfkampagnen darstellen [28].
